# The Influence of the Environment for Glass-Reinforced Plastic Composite Material Used for Ground Water Transport Pipes

**DOI:** 10.3390/ma14123160

**Published:** 2021-06-08

**Authors:** Ana Diana Ancaș, Corneliu Munteanu, Bogdan Istrate, Mihai Profire, Florin-Emilian Țurcanu

**Affiliations:** 1 Building Services Department, Faculty of Civil Engineering and Building Services, Gheorghe Asachi Technical University, 700050 Iaşi, Romania; ancas05@yahoo.com (A.D.A.); profiremihai@yahoo.com (M.P.); 2 Mechanical Engineering, Mechatronics and Robotics Department, Faculty of Mechanical Engineering, Gheorghe Asachi Technical University, 700050 Iaşi, Romania; cornelmun@gmail.com

**Keywords:** glass-reinforced plastic composite material, ground water transport pipes, pipe damage index, axial tension

## Abstract

Glass-reinforced plastic (GRP) composite materials are mainly used in the construction of pipes due to the wide range of sizes, ease of installation, adaptability to the specific situation in the field and, last but not least, the more competitive price as the nominal diameter increases. Their wide range of applications: drinking and raw water transport, sewerage, industrial waters, desalination plants, mining, etc., has led to the need to tailor the behaviour of the composite material to different fields, with pH values that are not neutral. Based on the experimental data, we aimed to study the change in the structure of the composite material as influenced by the soil characteristics: neutral, basic and acidic. In addition, starting with the pH of the three types of soil—basic, acidic and neutral—which significantly affect GRP composite materials, we calculated the pipe damage index and the Pearson correlation coefficients for axial tension. The results highlight the significant influence of the soil pH on the behaviour over time of the buried GRP pipes. Thus, laying the pipe in acidic soil significantly reduces its life, which should be taken into consideration during the design phase.

## 1. Introduction

Water transport pipes constructed from composite materials are relatively new. Thus, it is advantageous to extrapolate their behaviour throughout the long operating time to establish the trends that these pipelines follow in service. Behaviour comparisons between pipes constructed from composite materials and those constructed from classical materials (cast iron, concrete, steel) are also extremely useful in design in order to choose the most suitable solution for different situations [1,2].

Thus, it is necessary to conduct new studies to evaluate other aspects of the behaviour of composite pipes in different situations (effects generated by the nature and properties of the site, the impact of natural hazards such as landslides, floods, earthquakes, shocks, explosions, etc.) [3].

Glass-reinforced plastic materials are composites produced by creating alternate layers of fibreglass, resin, sand, quartz, dye, etc. They are used in various applications and fields, such as water and sewerage pipes, tanks, the automotive industry, aeronautics, petrochemicals, energy, sports products, construction, etc. The chemical composition of the material is at least 98% silicon dioxide (quartz sand), calcium alumina silicates (fibreglass) and thermally hardened resins [4].

The manufacturing technology consists of winding and centrifugation. The centrifugation technology uses only chopped glass wires, randomly arranged in the mass of the material, having the advantage of a higher density. Modern technology has enabled the highly compact lamination of the three materials, namely glass, sand and resin, thus optimizing the composition of the pipe according to the actual requirements in the field—both a circumferentially arranged continuous wire and a fibreglass shredder are used for increased axial strength. The addition of quartz sand increases the pipe wall thickness, thus removing the material from the neutral axis to increase the annular rigidity of the pipe. Therefore, three layers are created: the outer structural layer, the core and the lower structural layer [5,6].

For pressurised pipes or for those laid in trenches, the main challenge is the circumference, so the use of continuous wire rods in this context has unique advantages in operation. The main element of the technology is a rotating mould, with a length of 6 m; the length of each section is also limited [7,8]. Through a feed arm moving along the mould, it is subjected to various predetermined amounts (depending on the characteristics of the product pipe) of fibreglass shredder, resin and quartz sand while rotating at a relatively low speed. The quantities of the three components and the sequence of their delivery in the mould are controlled by a computer. After the final quantities are delivered to the mould, the rotational speed increases significantly in order to achieve the centrifugal force needed to ensure the desired degree of compression and ensure the dispersion of the materials according to the requirements. After cooling and stopping the rotational movement, the pipe is released from the mould, and the machined ends and the plug are added. The resistance structure consists of layers of resin and fibreglass, and the middle is filled with quartz sand mixed with resin. The application of these materials is performed continuously and successively, followed by heat treatment [9,10].

The standards for pipes constructed from fibreglass-reinforced polyester resins consider that the stresses to which the pipes are subjected are reflected in their mechanical deformation. Their projected lifespan is 50 years. Over the years, test results have demonstrated that their stability is much better than expected, leading to the notion that their lifespan can be extended to 150 years (in the case of Flowtite Gray Pipes, for example) [11,12].

Pipes constructed from composite materials, such as GRP, can be found in a wide range of corrosive environments, which can cause changes in the properties of the material.

The mass of the composite, due to the action of the underlying medium, and the phenomenon of modification of the structure of the material lead to its mechanical degradation [13,14,15,16,17].

Taking the above into consideration, it is necessary to adapt the material to the requirements of the particular environment in order to determine the type and the amount of fibreglass and resin to be used.

## 2. Materials and Methods

The resistance structure of GRP pipes consists of layers of resin and fibreglass, and the middle is filled with quartz sand mixed with resin. The application of these materials is performed continuously, in successive layers. After the formation of the pipeline, the heat treatment is applied, during which all the chemical processes are completed, and the length of the pipeline is established. The end is calibrated, and the plug is mounted.

Based on prior experience with water pipes, some damage has been attributed to errors in installation and problems relating to the minimum characteristics required for the filling material. Indeed, the pH of the soil also has an essential effect on the destruction of the pipe by degrading the mechanical characteristics of the GRP composite material. The experimental studies performed so far on GRP pipes have mainly focused on their behaviour when filled with substances, and less on the soil in which they are located. Regarding the influence of the soil on the composite material, many variables are involved; consequently, we used the Pearson correlation coefficient, developed by the English mathematician Karl Pearson at the beginning of the 20th century [18].

One of the previously completed studies [19,20] proposed a formula for quantifying the damage to the composite pipe applied to the flow load (the value of the force from which the behaviour of the pipe changes from elastic to rigid):

(1) $$D=1-\frac{F_{c}^{*}}{F_{c}}$$

where: *D*—the pipe damage parameter; $F_{c}^{*}$—break strength in basic and acidic soil;$\;F_{c}$—break strength in neutral ground.

The choice of the flow load for measuring the impact of pH on the axial behaviour of the composite material should take into consideration that, in practice, the load values will not equal the breaking load (due to the joining with plugs, possibly resulting in a plug-in in the case of a large axial load). In blocked or in laminated joints, although less commonly used (less than 3% of GRP pipes worldwide), the breaking load could be relevant in the short term [21,22].

We conducted the experimental study in acidic and basic soils based on these data. Neutral soil was used as control ground.

To highlight the influence of the terrain on the GRP pipes, we buried three sections of length 150 cm, DN250, wall thickness 6.2 mm, yield point 25 MPa, in three experimental locations.

The percentage composition was determined after calcination (JEPE) using the following values:

− Resin 26.41%;
− Fibreglass (CaAlSiO_8_)—38.28%;
− Sand (SiO_2_)—35.31%.

The first sample was taken from a petroleum field (the first experimental location), in the Zemeș area, Romania, which also contains water from a section of the main pipeline. The second sample was taken from a salt field (the second experimental location) in the Amara area of Romania, which was a basic soil.

For the third experiment, the control sample (neutral soil) was taken from an area where GRP pipes had been previously laid, which did not present problems in operation over time (Bacău area, Romania—the third experimental location).

These samples were analysed at the OSPA laboratory, Iași, Romania.

The method used was soil reaction, which is a measure of soil acidity or alkalinity. The soil pH (Table 1) was defined as the logarithm in base 10 of the activities of hydrogen ions (H^+^) or of hydronium ions (H_3_O^+^) in a solution. For simplification and standardization, a numerical scale from 0 to 14 was adopted. This size was considered the main variable of the soil. Moreover, its influence on the behaviour of GRP pipes was treated as a priority in this material. The principle of determining the soil reaction consists of extracting soluble forms from the soil in distilled water.

The humus and organic carbon content, the carbonate content and the soil solution composition were also determined. The method used was analysis by wet combustion of the soil using chromic acid.

Sample pipes constructed from GRP composite material used for buried water transport pipes were also buried in the three experimental locations. After 24 months, the samples were recovered; 3 specimens were extracted and evaluated, from a structural and morphological point of view, by electron microscopy and X-ray diffraction.

Morphological SEM (scanning electron microscopy) analyses were performed using the SEM FEI Quanta 200 3D (Brno, Czech Republic) equipment. Samples were scanned using an LFD (large field detector) detector with 20 kV high voltage at a working distance of 15 mm. XRD (X-ray diffraction) analysis was performed on the Xpert PRO MPD 3060 equipment, Panalytical (Almelo, The Netherlands), using a copper X-ray tube (Kα = 1.54051°), 2θ: 10°–80°.

An INSTRON 4400 universal test machine (Techincal University of Gheorghe Asachi from Jassy, Jassy, Romania) was used to determine the axial stress. A sample cut from the pipes was subjected to an increasing tensile force until it broke. The deformation and the respective force were measured and, taking into account the section, the mechanical and elastic characteristics were determined.

Based on the results obtained, we calculated the pipe damage index for axial tension and the Pearson coefficients for axial tension.

### Soil Characteristics Analysis

The results obtained from the tests performed at OSPA Iași, Romania are presented in Table 2, Table 3 and Table 4.

## 3. Results and Discussion

**Figure 1:**
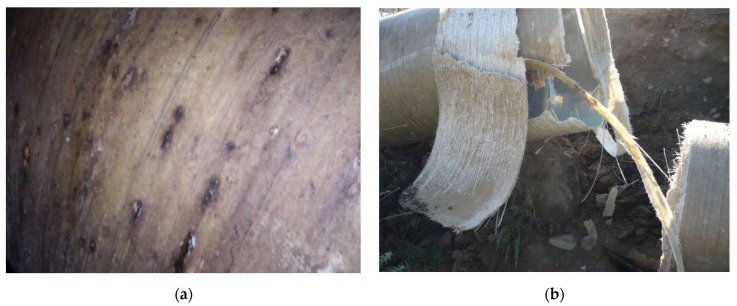
Damage to the glass-reinforced plastic pipeline in (**a**) basic soil and (**b**) acidic soil.

Figure 1 shows two photos taken at different locations along the pipeline: basic ground and acidic ground. In the case of the pipe buried in basic soil, exfiltration phenomena can be observed visually through the points of rust that appear at the level of the pipe, indicating the complete destruction of the pipe in the case of acidic ground.

In the case of acidic soil, the damage was manifested in a remarkable way. By changing the mechanical characteristics of acid-contaminated soils, their bearing capacity decreases. There is also a drastic alteration of the pipe material due to the significant effects on the glass fibre.

In the case of basic ground, leaks and colour changes could be observed in the pipe, without significant damage to the fibreglass.

### 3.1. SEM Analysis of GRP Materials

The evaluation of the modification of the GRP material structure was performed by analysing the areas related to the three characteristic layers of the pipe material: the outer structural layer, the core and the lower structural layer.

**Figure 2:**
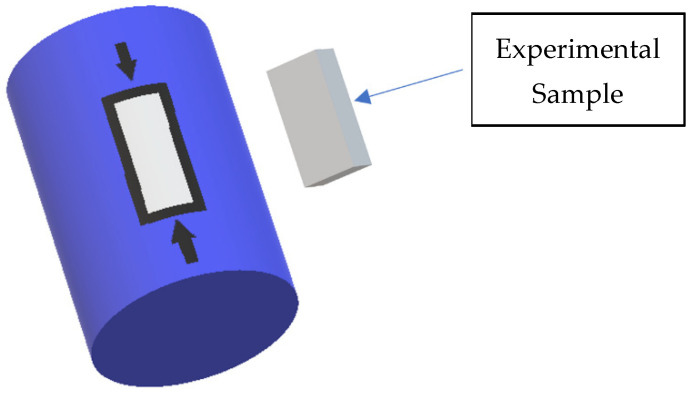
Sampling area for structural analysis.

For structural analysis using scanning electron microscopy, samples were taken according to Figure 2, and the specific analytical areas are highlighted by section in the longitudinal area of the pipe.

#### 3.1.1. Analysis of Changes in the Core of the Material over Time

**Figure 3:**
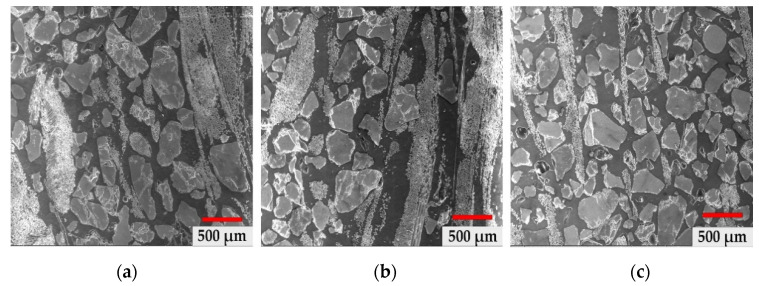
Morphological structure of the material core: (**a**) neutral soil, (**b**) basic soil, (**c**) acidic soil.

In Figure 3a–c, we have highlighted the structures in the core area of the GRP material at a magnification power of 100× for all three types of material samples collected from the three types of terrain. There is a structure with granulation and uneven distribution of glass particles and fibres, which is similar to the samples collected from neutral and basic terrain. In the acid field test, the structurally highlighted fibres were much smaller and thinner, which confirms the degradation of the glass fibres in the composite mass and thus the plasticization of the core structure in the acidic environment.

**Figure 4:**
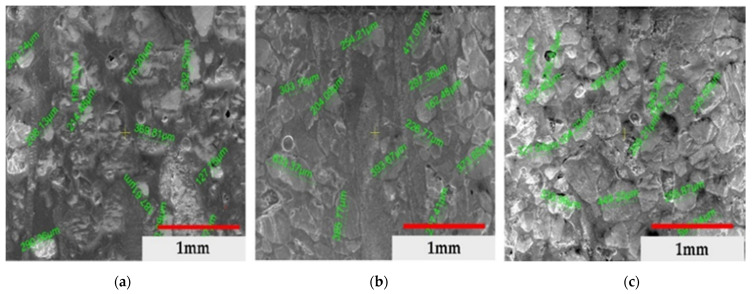
Average values of the particles: (**a**) neutral soil, (**b**) basic soil, (**c**) acidic soil.

Figure 3 shows the morphology of the component particles in the pipe in the specific medium (neutral, acidic and basic). There is a more accentuated fragmentation for samples a and c, respectively, in neutral and acidic environments, and a lower fragmentation for sample b, due to the presence of continuous fibres arranged in the longitudinal direction. The average dimensions of the particles have the following values (Figure 4): (a) neutral: 339.50 µm ± 146.63 µm, (b) basic: 321.58 µm ± 124.15 µm and (c) acid: 295.78 µm ± 111.58 µm.

#### 3.1.2. Analysis of Changes in the Outer Layer Thickness over Time

**Figure 5:**
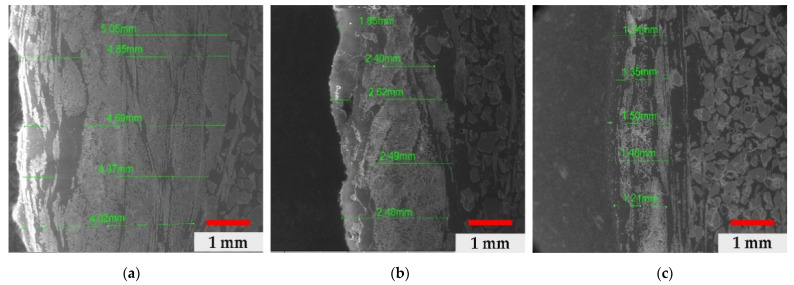
Dimensional variation in the outer layer: (**a**) neutral soil, (**b**) basic soil, (**c**) acidic soil.

From the analysis of the outer layer for the three cases—shown in Figure 5a–c—the fibrous structure obtained through the arrangement of glass fibres with different thicknesses is highlighted, with an average value of 4.608 mm for the neutral ground sample; an average value for 2.368 mm for the primary field sample and an average value of 1.34 mm for the acidic field sample. There is a reactive activity of the environment on the thickness of the outer layer, with a significant reduction in thickness by 50% in the case of basic soil and by 70% in the case of acidic soil.

**Figure 6:**
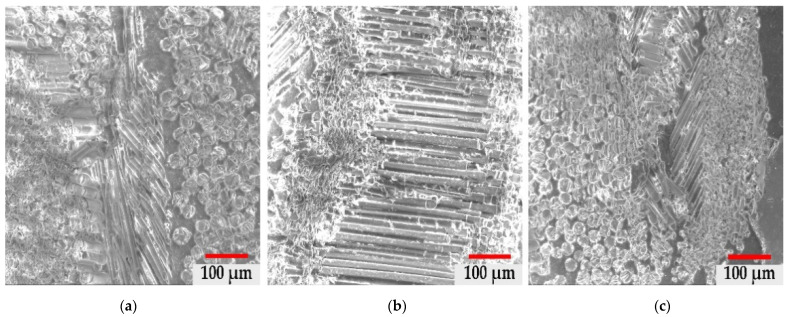
Morphological structure of the outer layer: (**a**) neutral soil, (**b**) basic soil, (**c**) acidic soil.

Structural analysis of external areas—as shown in Figure 6a–c—highlights fibrous structures with different orientations with the same calcium aluminosilicate morphology.

Figure 6 confirms the continuity of the fibres in the structure of the pipes used in a neutral environment, having a parallel and substantially equal fibre structure in different areas of the material and with different orientations, without a preferential orientation. We also highlighted the size of the component particles by extending the SEM analysis (Figure 4).

The mechanism of degradation of the pipe material differs from acidic soils with respect to neutral basic soils. In particular, in acid soils, there is a degradation of the outer layer by 70% as a result of increasing roughness, aging of the material, exfoliation, cracks and damage to the pipe.

The core of the material is negatively influenced by the pH of the soil, destroying the glass fibres and leading to plasticization of the material.

Fibreglass tends to undergo accelerated aging when exposed to an acidic solution; the fibres break, causing an overload of the rest of the fibres, thus causing major ruptures.

### 3.2. XRD Analysis of GRP Materials

**Figure 7:**
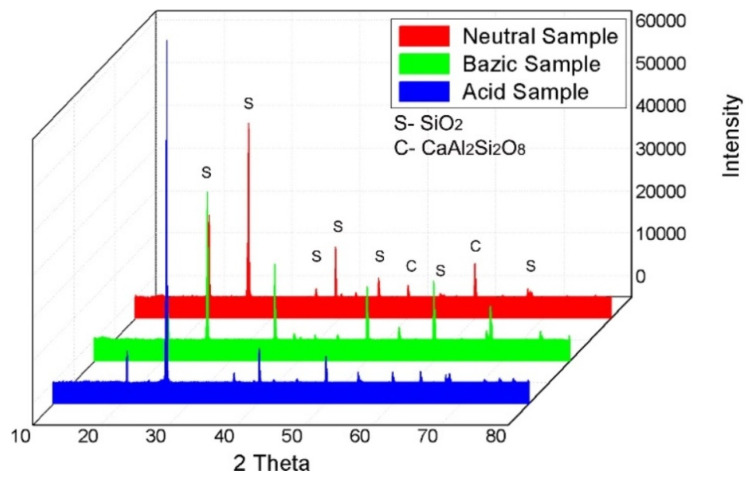
XRD analysis of 3 experimental samples.

Figure 7 shows the X-ray diffraction analysis results for the core of each of the three samples. The figure highlights the specific diffraction peaks of the two main components (SiO_2_ and CaAl_2_Si_2_O_8_).

Figure 7 shows the diffraction peaks for the component phases of the pipes used in the three different media. The figure presents the three cases in different colours and provides the related explanations.

**Figure 8:**
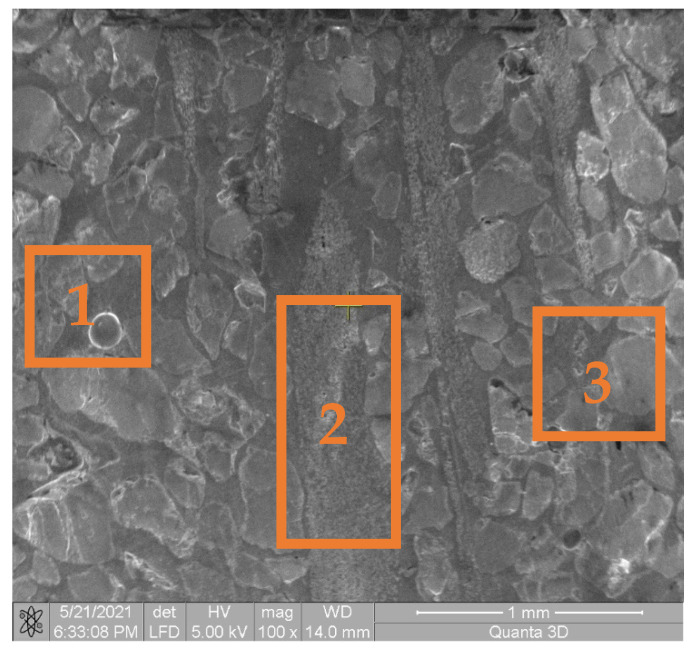
SEM images for different areas used for EDS chemical analysis. (1) SEM images for General aspect area, (2) SEM images for Continous Fibres area, (3) SEM images for Particles area.

EDS analysis of the component particles and base material was an important aspect of this study. The results are justified by the XRD analysis, in terms of the identified compounds. The diffraction peaks did not show displacements in terms of the 2θ angle, but there were clear differences in intensity for the acidic sample, which explains the higher crystallinity, Figure 8. The EDS results collected in three different areas are presented in Table 5.

### 3.3. Calculation of the Pipe Damage Index for Axial Tension

The values of the force from which the behaviour of the sample changes from elastic to rigid are as follows:

$F_{c}^{*}$ = 4.31 KN for basic soil;
$F_{c}^{*}$ = 3.99 KN for acidic soil;
$F_{c}^{*\;}$= 4.95 KN for neutral soil.

The corresponding result of the pipe damage index is:

$D=1-\frac{F_{c}^{*}}{F_{c}}=1-\frac{4.31}{4.95}=0.13=13\%$ for acidic soil;
$D=1-\frac{F_{c}^{*}}{F_{c}}=1-\frac{3.99}{4.95}=0.13=13\%$ for basic soil.

### 3.4. Calculation of Pearson Correlation Coefficients for Axial Tension

The average reference value for axial tension and standard deviations are presented in Table 6.

The results of the axial tensile test are presented in Table 7 for the correlation coefficients of pH and axial stress.

The measured values of Na (in me/100 g) related to the three fields, calculated using the Pearson correlation coefficients, yielded the following Pearson correlation matrix results (Table 8).

The low values of the pH correlation index (all other sizes) show a moderate influence of the pH variation on the mechanical characteristics. The only significant value (−0.342) shows the tendency for the load value at which the plastic deformation decreases slightly.

Positive correlation index values were calculated only between the concentration of Na ions and Young’s modulus (0.557), indicating the composite material’s tendency to plasticise. The relatively high value (−0.774) of the correlation index between the concentration of Na ions and the load at flow shows a general tendency to decrease the mechanical resistance in the axial direction with an increase in the basic content of the soil. Taken together, these two values present image of an aging phenomenon of the composite material, reflected especially by the change in the behaviour from semi-elastic to plastic.

## 4. Discussion

The evaluation of the structure of glass-reinforced plastic material in the outer layer and in the core indicates the degradation of the glass fibre in an acidic environment. The most used fibreglass is type E, with a high boron content, which gives it special mechanical properties but is affected by an acidic environment.

Scanning electron analysis presents a structure with granulation and uneven distribution of glass particles and fibres, a problem that is similar to the structures obtained from neutral and basic terrain. In the acid field test, fibres were found to be much smaller and thinner, which confirms the degradation of the glass fibres in the composite mass and thus the plasticization of the core structure in the acidic environment.

From the XRD point of view, the predominant peak is identified at the 2θ angle of 26.64° and is formed of silicon dioxide (COD: 96-710-3015) and has a crystalline structure of the hexagonal type. Secondary peaks consist of silicon dioxide and calcium aluminosilicate (COD: 96-154-0253), identified at various 2θ angles of 51.42° and 60.31°, having a crystalline structure of the tetragonal type. These results are in accordance with the structural and morphological analysis obtained by SEM analysis.

In the case of basic soil, the increase in the Young’s modulus in the axial direction is highlighted and the ability to work with the ground in the vicinity of the pipeline disappears; thus, the pipeline operates independently of the ground. However, due to the use of plugs for joining, which can be assimilated with elastic joints, the loss of elasticity in the axial direction is compensated.

From the point of view of the morphological structure of the material (Figure 2), it can be observed that it is more affected in the case of acidic soil than basic soil.

## 5. Conclusions

The results of our experimental study show that a unit deviation in pH in the acidic spectrum generates a decrease in the GRP pipe’s lifespan by 10 years. Thus, if the typical lifespan is 50 years for pipes buried in the ground with a neutral pH (pH = 7), at a pH value of 5, the lifespan will decrease to 40 years, and a pH value of 9 would further reduce the lifespan, from the perspective of stiffness depreciation.

In the case of the basic ground, although the morphological structure of the material is affected, the pipe does not lose its elastic character in the axial direction due to the joints, meaning that the service life is not affected.

In order to avoid the deterioration of the behaviour of the buried GRP pipes over time, a series of measures can be taken during the design phase; in particular, a petrochemical study is necessary to determine the pH of the soil solution at the required depth, as well as the presence of Na^+^, oil residues or other forms of pollution (anthropogenic or not).

In addition, the choice of a higher class of pressure and rigidity than the one obtained from the static calculation will lead to sufficient and long-term values of the mechanical characteristics for safety in operation, despite the steep regression curve of these characteristics over time.

From the microstructural analysis, it could be concluded that the samples presented a structure with granulation and uneven distribution of glass particles and fibres. In the acid field test, the microstructure displayed fibres that were much smaller and thinner than the other two media. During the XRD analysis, the predominant peaks were identified at the 2θ angle of 26.64° and were formed of silicon dioxide with a crystalline structure of the hexagonal type. Secondary peaks consisted of silicon dioxide and calcium aluminosilicate, identified at various 2θ angles of 51.42° and 60.31°, with a crystalline structure of the tetragonal type.

It would be advantageous to develop the concept of residual chemical stress, to introduce a factor for quantifying the structural degradation caused by chemical aggression on pipes constructed from composite materials.

## 6. Patents

This section is not mandatory but may be added if there are patents resulting from the work reported in this manuscript.

## Figures and Tables

**Table 1:** pH variation intervals depending on soil reaction.

Soil Reaction	pH Variation Intervals	Experimental Soil Type Perimeter
Strongly acidic	≤5	-
Moderately acidic	5.01–5.80	5.40
Weakly acidic	5.81–6.80	-
Neutral	6.81–7.20	6.90
Weakly basic	7.21–8.40	8.10
Moderately, strongly basic	≥8.40	-

**Table 2:** Acidic field results.

Soil Type	pH	P	P_corrected_	K	CaCO_3_	Humus	N_t_	Na^+^ tot	Na^+^ sol	Na^+^ comp	Ca^2+^	Mg^2+^
		ppm	ppm	ppm	%	%	%	me/100 g	me/100 g	me/100 g	me/100 g	me/100 g
acid	5.94	38.0	21.0	183.0	7.84	4.06	0.211	1.09	0.217	0.873	18.0	6.08

**Table 3:** Basic field results.

Soil Type	pH	P	P_corrected_	K	CaCO_3_	Humus	N_t_	Na^+^ tot	Na^+^ sol	Na^+^ comp	Ca^2+^	Mg^2+^
		ppm	ppm	ppm	%	%	%	me/100 g	me/100 g	me/100 g	me/100 g	me/100 g
basic	8.19	44.0	31.0	177.0	1.67	7.56	0.371	7.82	2.8	5.02	46.0	20.67

**Table 4:** Neutral field results (control field).

Soil Type	pH	P	P_corrected_	K	CaCO_3_	Humus	N_t_	Na^+^ tot	Na^+^ sol	Na^+^ comp	Ca^2+^	Mg^2+^
		ppm	ppm	ppm	%	%	%	me/100 g	me/100 g	me/100 g	me/100 g	me/100 g
neutral	7.03	21.0	10.0	157.0	3.50	1.20	0.068	0.87	0.87	0.217	20.0	6.08

**Table 5:** EDS chemical analysis.

EDS Chemical Analysis		%wt.C	%wt.O	%wt.Al	%wt.Si	%wt.Ca
General aspect	Neutral	28.89	37.41	0.61	28.08	5.01
	Basic	32.62	39.03	1.8	24.82	1.74
	Acidic	26.25	39.20	1.15	31.97	1.41
Continuous fibres	Neutral	34.37	32.74	3.47	18.46	10.96
	Basic	25.94	34.85	5.58	23.56	10.06
	Acidic	34.85	32.59	4.71	20.20	7.64
Particles	Neutral	8.58	47.89	0.42	43.60	0.50
	Basic	6.14	47.64	0.57	45.25	0.41
	Acidic	7.73	47.95	0.64	43.33	0.35

**Table 6:** Mean values of axial tension.

Variable	Neutral Soil	Standard Deviations	Basic Soil	Standard Deviations	Acidic Soil	Standard Deviations
Δ (%)	6.60	±0.07	4.70	±0.06	4.70	±0.06
F (KN)	4.95	±0.03	3.99	±0.06	4.31	±0.05
E (MPa)	672.00	±3.26	852.00	±4.66	844.00	±4.05
σ_r_ (MPa)	46.38	±0.08	40.50	±0.14	39.45	±0.07
σ_max_ (MPa)	46.40	±0.11	40.50	±0.16	39.45	±0.09

Δ—maximum deformation; F—break strength; E—Young’s modulus; σ_r_—failure strain; σ_max_—maximum stress.

**Table 7:** pH–axial stress correlation coefficients (Pearson correlation matrix).

Variable	pH	Δ	F	E	σ_r_	σ_max_
pH	1	−0.015	−0.342	0.055	0.125	0.125
Δ	−0.015	1	0.945	−0.999	0.990	0.990
F	−0.342	0.945	1	−0.957	0.890	0.890
E	0.055	−0.999	−0.957	1	−0.984	−0.984
σ_r_	0.125	0.990	0.890	−0.984	1	1000
σ_max_	0.125	0.990	0.890	−0.984	1000	1

Δ—maximum deformation; F—break strength; E—Young’s modulus; σ_r_—failure strain; σ_max_—maximum stress.

**Table 8:** Na^+^ correlation coefficients–axial effort (Pearson correlation matrix).

Variable	Na^+^	Δ	F	E	σ_r_	σ_max_
Na^+^ (me/100 g)	1	−0.524	−0.774	0.557	−0.399	−0.399
Δ	−0.524	1	0.945	−0.999	0.990	0.990
F	−0.774	0.945	1	−0.957	0.890	0.890
E	0.557	−0.999	−0.957	1	−0.984	−0.984
σ_r_	−0.399	0.990	0.890	−0.984	1	1000
σ_max_	−0.399	0.990	0.890	−0.984	1000	1

Δ—maximum deformation; F—break strength; E—Young’s modulus; σ_r_—failure strain; σ_max_—maximum stress.

## Data Availability

The data underlying this article will be shared on reasonable request from the corresponding author.
